# Health behaviors among head and neck cancer survivors

**DOI:** 10.1186/s41043-023-00390-6

**Published:** 2023-05-29

**Authors:** Erin McMenamin, Abigail Blauch Gottschalk, Donna A. Pucci, Linda A. Jacobs

**Affiliations:** Abramson Cancer Center, Perelman Center for Advanced Medicine, University of Pennsylvania, South Tower 10-149, 3400 Civic Center Boulevard, Philadelphia, PA 19104 USA

**Keywords:** Head and neck cancer, Cancer survivorship, Health behaviors, Human papilloma virus

## Abstract

**Purpose:**

To determine to what extent head and neck cancer (HNC) survivors participate in health behaviors (HBs) recommended by the National Cancer Center Network (NCCN®).

**Methods:**

Participants identified through the tumor registries at the Abramson Cancer Center (ACC), University of Pennsylvania and affiliated sites. Eligibility: (a) diagnosis and treatment HNC; (b) aged 18 to 70 years; (c) ≥ 1-year post-diagnosis; (d) human papillomavirus (HPV) status confirmed; (e) ability to understand written English. Potential participants received an explanation of the study, informed consent, self-reported questionnaire, and self-addressed stamped envelope.

**Results:**

451 individuals eligible, 102 (23%) agreed to participate, HPV positive (74%). Current smoking rare (7%), historical use common (48%). Current alcohol use common (65%), average 2.1 drinks/day, 12 days/month. 22% binge drank with an average of 3.5 binge-drinking sessions per month. Nutritional behavior mean 7.1 (range 0–16), lower scores indicating better nutrition. Body mass index (BMI) 59% overweight/obese. Adequate aerobic exercise 59%, adequate strength and flexibility 64%. Leisure time activity, 18% sedentary, 19% moderately active, 64% active. All participants reported having a primary care physician, 92% seen in the previous 12 months.

**Conclusions:**

Most HNC survivors participated in some HBs. Current smoking rarely reported, binge drinking and high BMI most common negative HBs. Opportunities remain to improve dietary and exercise behaviors.

**Implications for cancer survivors:**

The NCCN® has outlined HBs that decrease likelihood of cancer survivors developing comorbidities that could impact overall survival. It is incumbent on healthcare providers to educate and encourage cancer survivors to participate in these HBs.

## Introduction

HNC accounts for almost 4% of cancers in the United States (U.S.) and 3% worldwide [1, 2]. Substantial declines in smoking prevalence have occurred over the past five decades in all age groups after the surgeon general’s report associated smoking with the development of cancer [2]. However, in 2021 according to the ACS, cigarette smoking continued to account for close to 3 out of the 10 million healthy life years lost to cancer per year in the U.S. Close to three quarters of non-HPV associated HNCs are associated with tobacco and alcohol use [3]. The risk of HNC also rises with the increasing number of alcoholic drinks per day and the duration of drinking, for oral cavity, hypopharynx, larynx, and highest for oropharynx cancers [4, 5]. Other behavioral factors such as dental hygiene, diet, activity level, and body mass index (BMI) are also associated with HNC incidence [6–10]. In fact, oral health impacts the risk for and survival of HNC [11, 12].

The impact of dental health on survival in HNC is greater for oropharynx cancer patients compared to other sites in the head and neck, and in contrast to previous studies, this was independent of education and income [13]. Whether poor oral health increases the risk of HPV-related oropharynx cancer is inconclusive to date [14].

While the rate of non-HPV related HNC is decreasing due to decreased smoking prevalence, the incidence of HPV related cancers is increasing at a rate of approximately 2.5% per year [15], and HPV accounts for approximately 70% of oral cancers [3]. HPV-related HNC is primarily a result of HPV infection contracted through sexual behavior [2, 3, 16].

The prognosis for oropharynx cancer is dependent on four factors: HPV status, pack years of tobacco smoking, tumor stage, and nodal stage [17]. HPV-related cancer of the oropharynx has a higher 3-year survival rate compared to HPV-negative cancer (82.45 vs. 57.1%) [17]. Factors other than HB explain a portion of the increased survival in HPV related HNC. These factors include but are not limited to decreased morbidity due to decreased alcohol and tobacco use, the increased sensitivity oof HPV related tumors to radiation and chemotherapy, as well as a possibly a lower risk for other cancers due to the decreased amount and duration of smoking and alcohol consumption in the population [18].

Recent evidence suggests HPV infection increases the risk of other oral cancers, including tongue, palate, and floor of the mouth [19]. Although pretreatment HBs such as tobacco use, diet, and physical activity level predict survival among HNC patients, little is understood about the HBs of survivors of HNC. What is known regarding HBs primarily focuses on tobacco and alcohol use among non-HPV associated HNC survivors [12, 18, 19]. In addition, very little is known about the HBs of individuals with HPV-associated HNC, an unfortunate gap in our ability to plan effective interventions for improving both survival and quality of life of this growing subpopulation of cancer survivors.

Several HBs are problematic for HNC patients. More than a quarter screen positive for problem drinking or alcoholism at diagnosis [20–22]. Between 25 and 50% of HNC patients are smoking at diagnosis [24–26], and up to 90% have at least a history of tobacco use [27]. Although less is known about cessation, studies have suggested that up to 84% of this population quit smoking spontaneously [20] around diagnosis, though the relapse rate can range upward of 60% for those smoking in the week prior to surgery [21]. Duffy [24] found that in the initial year following treatment there were reductions in smoking and problem drinking behaviors (to 21% and 11%, respectively), and physical activity rebounded; however, sleep remained particularly poor.

Data also suggests that HNC patients have multiple HB deficits, including poor intake of fruits and vegetables [24]. In addition, efforts to examine physical activity have been few and included individuals on treatment, limiting generalizability to survivors [26]. It appears that immediately following treatment HNC survivors reduce their physical activity [27–29] and recreational activity by more than 60% [30] and many experience severe weight loss [31]. These are important since HBs such as smoking and maintenance of weight and physical activity are related to psychosocial and fatigue outcomes [31, 32], and HBs are associated with both IL-6 levels and survival in HNC patients [12, 24].

Little is known about post-treatment HBs among HPV associated HNC survivors, including the prevalence of tobacco and alcohol use in patients with HPV associated HNC. There are also a number of HBs, such as dental care, diet, and adherence to cancer and non-cancer surveillance recommendations for which little is known for HNC survivors regardless of HPV status. This is of particular importance since HNC comorbidities (e.g., cardiac and respiratory issues) are likely to be at least partly moderated by HBs of these individuals.

The goal of this study was to describe modifiable determinates of HBs and illness perceptions among HNC survivors to inform interventions to improve long-term outcomes. Moreover, we recognized that these HBs and perceptions might differ according to HPV status and other factors which have been shown to influence HBs among cancer survivors.

### Objectives

The primary objective of this study was to describe the range of HBs relevant to both recurrence and survivorship among HNC survivors and determine whether these differ by HPV status and demographics. In addition, measures of illness QoL and distress among HNC survivors were included to determine whether these differ by HPV status and demographics.

Lastly, assessment of the relationship between illness perception, QoL, distress, and HB among HNC survivors was classified by HPV genotype status to determine if population stratification is necessary in intervention design.

## Methods

Potential participants were identified and screened by our research personnel through the tumor registry at the Abramson Cancer Center (ACC) of the University of Pennsylvania as well as affiliated sites (Chester County Hospital, Lancaster General Hospital, Pennsylvania Hospital, Penn Presbyterian Medical Center) to allow for broader reach. Potential participants were eligible if they: (a) had a diagnosis and at least partial treatment of HNC as reported in the registry; (b) were between 18 and 70 years of age; (c) were more than 1-year post-diagnosis; (d) had a recorded confirmation of HPV status; and e) could understand written English.

Potential participants were contacted via mail with an opt out option. Those who did not opt out were contacted by phone or email and asked to participate in a study. If potential participants were unavailable by telephone after two attempts, staff contacted them by mail. Potential participants received an explanation of the study, informed consent documentation, a self-reported questionnaire, and a self-addressed stamped envelope for return of materials. Individuals not returning the materials within the first three weeks received up to two additional phones calls to encourage completion and return of the study materials. This process was repeated on an ongoing, weekly basis. Once data were received, they were entered into a secured database.

### Measures

A few specific instruments make up the self-reported questionnaire.

Demographic and Medical Variables – All participants responded to standard demographic questionnaires assessing age, ethnic/racial identification, marital status, number of offspring, educational level, income, insurance status, living situation, and presence of chronic medical comorbidities.

Health Behaviors – HBs included items recommended by Glasgow et al. assessing aerobic activity, strength-flexibility, leisure time activity, smoking, alcohol consumption, and access to a physician [4]. This battery assesses physical activity levels using the RAPA and the Godin Leisure Time Activity Scale [33]. Drinking behavior was assessed using items from the BRFSS [34] and cigarette smoking with three items from national health surveys which query smoking history, current smoking status, and extent of smoking. Eating patterns were assessed with items from the STC-Diet [35] that assesses food patterns vs. nutrient or fat intake. We assessed cancer and non-cancer health surveillance behaviors using tools developed for other, similar applications.

Illness Perceptions – The Illness Perception Questionnaire-Revised (IPQ-R) [36] was used to quantitatively assess attributes of illness. The IPQ-R is a self-report, 84-item questionnaire. It assesses illness identity causality, consequences, timeline, control/cure attributions, illness coherence, and emotional representations, and keyed specifically to the HNC illness experience.

Psychological Distress – We assessed cancer-specific distress using the Impact of Event scale (IES) [37], which has been widely used in a variety of cancer patient and survivor populations. Past studies report acceptable internal consistency and discrimination between situations of varying stressfulness. Instructions for the IES key responses to specific experiences for this study was the HNC experience.

Quality of Life – We assessed QoL in two ways. The University of Washington QoL Questionnaire (UW-QOL) [38] is a brief assessment of 12 individual domains (activity, appearance, chewing, dry mouth, employment, pain, recreation, saliva, shoulder function, speech, swallowing, and taste) relevant to HNC survivors. It also allows for more global ratings of QoL, has been used in numerous examinations of HNC outcomes [39, 40], and non-cancer normative values are available. The RAND Medical Outcomes Study Short-Form-36 Health Survey (SF-36) [41] was used to assess emotional and social functioning and role limitations. This measure has been used extensively among various medical populations, yielding rich normative data [42–45]. The psychometric properties of the measure are well-established [46–50], and it has been used to demonstrate validate several QoL instruments [51].

### Statistical analyses and results

Medical record abstraction included patient demographics, primary treatment, tumor site, and HPV status.

Analysis was restricted to enrolled patients who returned surveys.

A total of 905 individuals were initially screened for eligibility. Of these, 451 were eligible and approached. A total of 102 (23%) patients participated in the study, and usable data were collected from 93 participants. Table 1. presents the demographic characteristics of the sample.

**Table 1:** Sample characteristics

Sample Characteristic (*n* = 93)	M (SD)	Range
Age	59.2 (7.8)	28–70
Time since diagnosis (yrs)	3.6 (2.6)	1–16
*N (Percentage)*		
White race	89 (96%)	
Female gender	19 (20%)	
Married or similar status	76 (82%)	
*Income*		
Less than $60,000 yr	16 (18%)	
> $60,000 yr	74 (82%)	
*Education*		
Less than college graduate	34 (37%)	
College graduate or above	59 (63%)	
*BMI*		
Underweight	2 (2%)	
Normal	36 (39%)	
Overweight	39 (48%)	
Obese	15 (16%)	
*Stage at diagnosis*		
1–2	15 (16%)	
3	9 (10%)	
4	51 (55%)	
DK	18 (19%)	
Surgery Chemotherapy Radiation Cancer relapse Second cancer HPV positive	67 (72%) 49 (53%) 81 (87%) 6 (7%) 18 (19%) 69 (74%)	
Current smoker	6 (7%)	
Past smoker	43 (48%)	
Current ETOH use	60 (65%)	

As can be seen in Table 1, the sample was predominantly white, middle, aged, married, well-educated, and well-resourced. Indeed, fully 63% of the sample reported an annual household income of greater than $100,000. Medical record review found that almost 3/4 of the sample were HPV positive.

### Results for aim 1

Describe the range of HBs relevant to both recurrence and survivorship among HNC survivors and determine whether these differ by HPV status and demographics.

Smoking and Alcohol use. Although current smoking was rare (7%), historical use of tobacco was common (48%). Current alcohol use was also common (65%). Among those who reported alcohol use, there was an average of 12 days per month in which alcohol was consumed, though this ranged from 1 to 30 days. On those days in which alcohol was consumed participants reported an average of 2.1 drinks per day. Binge drinking (consuming > 5 drinks in one sitting) was reported by 22% of those who drank, with an average of 3.5 binge drinking sessions per month among those reporting any binge drinking. There was no relationship between HPV and smoking status. Women were more likely than men, however, to be current smokers (*p* < 0.05). There was no relationship between current alcohol use status, days drinking per month, or number of drinks on average per drinking session and either HPV status or gender.

Diet and Weight. Scores on Starting the Conversation—Diet (SCT-Diet), our measure of nutritional behavior can range from 0 to 16 with lower scores indicating better nutrition. Overall, the sample mean was 7.1 (SD = 2.4) with scores ranging from 0 to 13. BMI was calculated from self-reported height and weight. Forty-one percent of the overall sample were Normal or Underweight, while 59% were overweight or obese.

There was no relationship between HPV status or gender with respect to nutritional behavior. Men had a higher BMI (M = 27.3) than women (24.0; F = 7.01, *p* < 0.01), and were more likely to be obese (*p* < 0.05).

Physical Activity. With respect to the overall sample, 59% reported adequate aerobic exercise and 64% adequate strength and flexibility on the RAPA. With respect to leisure time activity, 18% would be classified as sedentary, 19% as moderately active, and 64% as active. Individuals with HPV positive status were marginally more likely to meet criteria for adequate strength and flexibility than those with negative status (*p* = 0.07), although there was no difference with respect to HPV status for aerobic activity or leisure time activity status. Men were more likely than women to report adequate aerobic activity (*p* < 0.01), though there was no gender difference for strength and flexibility or leisure time activity status.

Medical Health Behaviors. All participants reported having a current primary care physician and 92% reported having had a physical examination in the previous 12 months, precluding examination of differences.

### Results for aim 2

Describe QoL and distress outcomes among HNC survivors and determine whether these differ by HPV status and demographics.

Quality of Life. Table 2. presents the overall mean scores for the SF-36 subscales assessing general health-related QoL as well as normative values from a sample of US. QoL on the SF-36 is similar to population values for Role Limitations due to physical functioning, social functioning, mental health, and role limitations due to mental functioning. HNC survivors reported higher physical functioning, less bodily pain, but lower vitality and general health than normative values. There were no significant differences between groups based on HPV status. Compared to men, women reported significantly more Bodily Pain, and greater disruption of Physical Functioning, Role Limitations due to Physical Functioning, and Social Functioning (all *p* < 0.05).

**Table 2:** SF-36 values and normative values

Domain	Mean (SD)	Normative value
Physical functioning	90.1 (14.3)	84.2 (23.3)*
Role limitations (Physical)	80.2 (34.5)	81.0 (34.0)
Bodily pain	82.4 (21.1)	75.2 (23.7)*
Social functioning	83.3 (25.4)	83.3(22.7)
Mental health	74.3 (18.6)	74.7 (18.1)
Role limitations (Mental)	81.9 (34.4)	81.3 (33.0)
Vitality	51.7 (10.9)	60.9 (21.0)*
General health	65.4 (20.0)	72.0 (20.3)*

^*^ *p* < 0.01

With respect to the HNC cancer specific UW-QOL measure, composite scores reflecting Physical and Social Functioning were computed. Scores can range from 0 to 100 with higher values reflecting better QoL. Physical Functioning was high (M = 80.5, SD = 13.7) and significantly higher (*p* < 0.001) than the normative value of 71 found among a large sample of similar patients. Similarly, Social Functioning was high (M = 79.1, SD = 16.9) and significantly higher than the normative value of 74 (*p* < 0.01). There were no significant differences between groups based on HPV status. Males reported significantly higher Physical (M = 82.1 vs. M = 72.9; *p* < 0.05) and Social Functioning (M = 81.9 vs. M = 67.8; *p* < 0.05) compared to females.

Cancer Specific Distress. Scores on the IES were computed for Intrusion and Avoidance subscales as well as the overall composite which was then used to categorize level of distress. Table 3. presents the categorical data. There were no significant differences between groups based on HPV status. Females reported significantly higher Intrusion (M = 11.9 vs. M = 6.0; *p* < 0.01) Avoidance (M = 13.1 vs. M = 6.6; *p* < 0.01) and overall cancer-specific distress (M = 25.0 vs. M = 12.6; *p* < 0.01) compared to males.

**Table 3:** Cancer specific distress

Category	*N* (%)
No distress	45 (50%)
Mild distress	24 (26%)
Moderate distress	16 (18%)
Severe distress	6 (7%)

### Results for aim 3

Describe illness perceptions underlying cancer and non-cancer future events and determine whether these differ by HPV status and demographics.

The IPQ-R assesses the common sense model illness perceptions: Identity (symptoms associated with the disorder), timeline (the expected trajectory or cyclical nature of the disorder), consequences (the anticipated outcome of the disorder), and treatment and personal controllability (what can be done to control the threat posed by the disorder and by whom), illness coherence (an individual’s sense that their illness representation is coherent and useful), and emotional representation (negative affective reactions to the illness). Higher scores in the dimensions including identity, timeline, consequences, and emotional representation reflect more strongly held beliefs about the number of symptoms attributed to the illness, the chronicity of the condition, the negative consequences of the illness, and negative emotional reactions to the illness. High scores on the personal control, treatment control and coherence dimensions, represent positive beliefs about the controllability of the illness and a personal understanding of the condition. See Table 4. for the overall group descriptive statistics for the illness perception variables.

**Table 4:** CSM illness perceptions

Domain	Mean (SD)
Identity	3.2 (3.1)
Timeline chronicity	16.2 (6.5)
Timeline cyclical	9.0 (3.3)
Consequences	18.8 (5.4)
Personal control	20.8 (4.7)
Treatment control	18.8 (3.2)
Illness coherence	19.3 (3.9)
Emotional representation	16.1 (5.6)

With respect to HPV status, individuals with non-HPV related HNC reported significantly stronger beliefs in the chronicity of their condition than individuals with HPV related disease (M = 18.4 vs. M = 15.4, *p* < 0.05). No other differences were noted between HPV status groups. There were no significant differences between groups as a function of gender.

### Results for aim 4

Assess the relationship between illness perception, QoL, distress, and HB among HNC survivors classified by HPV status, and determine if population stratification is necessary in intervention design. Unfortunately, the low prevalence of non-HPV related HNC and few females make stratification by these variables for the following analyses highly unreliable due to power considerations and risk for overfitting of models. Thus, only overall group relationships were examined and reported.

Table 5. presents the correlations between illness perception variables and HBs. As can be seen, illness perceptions appear remarkably unrelated to HBs among HNC survivors with the exception of Personal Control beliefs which are better dietary behaviors and greater leisure time activity.

**Table 5:** Correlations between illness perception variables and HBs

	Aerobic	Strength & flexibility	Diet	Leisure time activity	BMI
Identity	− .069	0.158	− 0.016	0.056	− 0.143
Timeline (Chronic)	− .013	− .0041	− 0.064	0.019	− 0.118
Timeline (Cyclical)	0.017	− 0.099	− 0.001	− 0.023	0.095
Consequences	− 0.017	0.062	− 0.066	−0.029	− 0.058
Personal control	.136	.182	− 0.295^**^	0.236^*^	− 0.063
Treatment control	− .117	.180	− 0.167	− 0.084	0.036
Illness coherence	− .059	.041	0.160	0.105	0.065
Emotional representation	− .033	− .095	− 0.111	− 0.046	− 0.109

^**^ Correlation is significant at the 0.01 level. * Correlation is significant at the 0.05 level

Table 6. presents the correlations between illness perception variables and QoL as assessed by the SF-36. The results demonstrate illness perceptions that relate significantly and moderately to many aspects of QoL. Identity, expected chronicity, negative consequences, and a negative emotional representation of HNC relate negatively to all aspects of QoL. The degree to which HNC is seen as cyclical relates negatively to all QoL domains except physical functioning, while the degree to which the illness makes sense to the individual relates positively to these domains. A sense of personal control over the disease and its treatment result(ed) in better QoL in all areas except physical functioning and limitations due to emotional function. Treatment control also was unrelated to limitations due to physical functioning.

**Table 6:** Correlations between illness perception variables and QoL as assessed by the SF-36

	Physical function	Social function	Limited physical	Limited emotional	Emotional wellbeing	Vitality	General health
Identity	− 0.27^*^	− 0.42^**^	− 0.45^**^	− 0.41^**^	− 0.42^**^	− 0.28^**^	− 0.31^**^
Timeline (Chronic)	− 0.22^*^	− 0.53^**^	− 0.36^**^	− 0.37^**^	− 0.34^**^	− 0.33^**^	− 0.45^**^
Timeline (Cyclical)	− 0.07	− 0.34^**^	− 0.22^*^	− 0.34^**^	− 0.33^**^	− .027^*^	− 0.33^**^
Consequences	− 0.36^**^	− 0.48^**^	− 0.48^**^	− 0.40^**^	− 0.44^**^	− 0.37^**^	− 0.30^**^
Personal control	.08	0.28^**^	0.22^*^	0.18	0.31^**^	0.34^**^	0.31^**^
Treatment control	− 0.01	0.41^**^	0.11	0.20	0.26^*^	0.35^**^	0.29^**^
Illness coherence	0.06	0.32^**^	0.22^*^	0.40^**^	0.40^**^	0.23^*^	0.33^**^
Emotional representation	− 0.22^*^	1− 0.44^**^	− 0.41^**^	− 0.45^**^	− 0.51^**^	− 0.43^**^	− 0.35^**^

^*^Correlation is significant at the 0.05 level. ** Correlation is significant at the 0.01 level

Table 7. shows the relationship between illness perceptions and HNC specific QoL on the UW-QoL measure. All illness perceptions were associated with HNC-specific QoL. Increasing beliefs in identity, chronicity and cyclical nature of the disease timeline, negative consequence, and negative emotional representation were related to worse physical and social QoL. A sense that one could exert control over the disease or its treatment and that one could make sense of HNC were related to better physical and social QoL.

**Table 7:** Correlations between illness perceptions and HNC specific QoL

	Physical QoL	Social Qol
Identity	− 0.49**	− 0.55^**^
Timeline (Chronic)	− 0.51*^*^	− 0.56^**^
Timeline (Cyclical)	− 0.25*	− 0.36^**^
Consequences	− 0.54^**^	− 0.52^**^
Personal control	0.41**	0.41^**^
Treatment control	0.45**	0.43^**^
Illness coherence	0.36**	0.35^**^
Emotional representation	−0 .46^*^	− 0.52^**^

^*^ Correlation is significant at the 0.05 level. ** Correlation is significant at the 0.01 level

Table 8 shows the correlations between illness perceptions and cancer-specific distress as assessed by the IES total score. Results are identical for the IES Intrusion and Avoidance subscales. Increasing beliefs in Identity, chronicity and cyclical nature of the disease timeline, negative consequence, and negative emotional representation were related to more cancer-specific distress. A sense that one could exert control over the disease and that one could make sense of HNC were related to decreased cancer-specific distress.

**Table 8:** Correlations between illness perceptions and cancer-specific distress as assessed by the IES total score

	IES-Total
Identity	0.58**
Timeline (Chronic)	0.36**
Timeline (Cyclical)	0.31*
Consequences	0.46^**^
Personal control	− 0.22**
Treatment control	− 0.15
Illness coherence	− 0.35**
Emotional representation	0.58^*^

* Correlation is significant at the 0.05 level. ** Correlation is significant at the 0.01 level.

Table 9. presents the correlations between HBs and indices of general QoL, HNC-specific QoL, and Distress. Among HBs, only aerobic exercise activity was associated with QoL. In this case, greater aerobic activity was associated with better physical functioning, fewer physical limitations, greater vitality, better general health, and better cancer specific social QoL. No HBs were associated with cancer-specific distress.

**Table 9:** Correlations between HBs and indices of general QoL, HNC-specific QoL, and Distress

	Physical function	Social function	Limited physical	Limited emotional	Emotional wellbeing	Vitality	General health	Physical QoL	Social QoL	IES total
Aerobic	0.32^**^	− 0.07	0.22^*^	− 0.03	0.11	0.26^*^	0.28^**^	0.02	0.22^*^	− 0.07
Strength-flexibility	− 0.00	− 0.03	− 0.13	− 0.07	0.10	0.09	0.10	− 0.02	0.00	0.08
Diet	0.07	0.02	− 0.11	− 0.02	− 0.12	− 0.19	− 0.12	0.02	− 0.08	− 0.13
LT activity	0.21	− 0.07	0.07	− 0.05	0.07	0.13	0.16	− 0.09	0.09	− 0.01
BMI	− 0.11	0.10	0.09	0.08	0.07	− 0.01	− 0.11	0.19	0.10	− 0.15

^**^Correlation is significant at the 00.01 level (2-tailed). * Correlation is significant at the 0.05 level (2-tailed)

## Discussion

The NCCN recommends several healthy behaviors for cancer survivors as they are associated with improved cancer treatment outcomes, QoL, and decreased comorbidity [52, 53]. The behaviors include maintaining a healthy weight, including exercise in their life, eating a healthy diet, smoking cessation, limiting alcohol intake, engaging in safe sun exposure, obtaining adequate amounts of sleep, and seeing their primary care provider (PCP) annually [52]. A previous study by Hyland et al. [54] found only 7.6% of cancer survivors participated in all recommended behaviors.

In general, participants in this study participated in the HB described at a higher rate than the previously noted literature. However, most subjects participated in some, but not all HBs, consistent with previous literature. Further investigation in future trials could determine whether this is related to the demographic composition of the participants and/or other factors.

Clark et al. [55] reports decreased self-management behaviors, functional well-being, and health related QoL (HRQOL) in HNC survivors with low health literacy. Low health literacy was highly associated with lower education level as well as living alone in their study. Walters et al. [56] reviewed interventions to improve health literacy and found that many studies reported successful interventions leading to improvement health literacy and self-management behaviors in the intervention groups. The studies were, however, conducted for various diagnoses other than cancer and had a high or serious risk of bias calling the results into question. Hoyle et al. [57] found that patients who were further out from treatment (> 5 years), who lived a distance to the clinic, and were unmarried were more likely to be lost to follow up after treatment for HNC.

The European Organization for Research and Treatment of Cancer (EORTC) previously established a symptom grading tool for patients who receive treatment for cancer, tracking the severity (grade) of symptoms. The EORTC is now developing a QoL tool investigating health behaviors as well as physical, psychological, and social aspects of HRQOL to further capture the impact of treatment on the cancer survivor [58]. This information has the potential to provide a greater understanding of the long-term impact of treatment-related symptoms on cancer survivors.

Osazuwa-Peters et al. [59] reported that the suicide rate for HNC survivors is almost 2 times higher than survivors of other cancers. Assessment for referral to mental health resources is an essential component of cancer treatment and survivorship, regardless of location. These services may be more accessible in many settings with the availability of telehealth. Cancer survivor support groups may also be a source of support for survivors.

No significant reports of depressed mood or suicidal ideation were identified in trial participants. HPV-related cancer has improved long-term survival compared to HPV non-related cancer. Improved surgical and radiation techniques have more recently led to the potential for fewer and/or less severe long-term toxicities and improved QoL. Further study is needed regarding if and how these issues impact the suicide rate.

Web based resources are a potential source of support for patients and their families before, during and after treatment for HNC. Fang et al., [60] reported the use of web-based resources to assist patient with symptom management and preventive care for the cancer survivor. Other sites include information regarding the cancer, treatment methods, side effects and management, as well as survivorship issues.

### Strengths and limitations

The participants provided information regarding a wide range of physical, psychological, and general well-being issues impacted by their cancer in this trial. After completion of treatment, a portion of survivors in this study continued to participate in behaviors that place them at risk for additional cancers. These results provide insight into areas of focus for potential future educational issues aimed at patients undergoing treatment for HNC.

A limitation of this study is the small number of non-HPV + participants. This is the result of the study being conducted primarily in an urban academic center as well as the fact that the HPV + HNC is outpacing non-HPV HNC diagnoses. Potential future studies could focus on the participation of multiple centers to capture a more heterogeneous population. Although the results of this study are not generalizable to all HNC patients, they are generalizable to the growing HPV^+^ HNC population. [61, 62].

## Conclusions

HBs that are important for cancer survivors have been identified by the NCCN. Most participants in this study participated is some of the recommended HBs after completing their cancer treatment. Regrettably, several HNC survivors continued to participate in risky behaviors, placing them at risk for recurrence and/or other cancers. Future research should include educational interventions during treatment as well as during survivorship to provide a better understanding of the importance of following the recommended HBs to maintain optimal health and well-being.
